# Magnolin inhibits cell migration and invasion by targeting the ERKs/RSK2 signaling pathway

**DOI:** 10.1186/s12885-015-1580-7

**Published:** 2015-08-08

**Authors:** Cheol-Jung Lee, Mee-Hyun Lee, Sun-Mi Yoo, Kyung-Il Choi, Ji-Hong Song, Jeong-Hoon Jang, Sei-Ryang Oh, Hyung-Won Ryu, Hye-Suk Lee, Young-Joon Surh, Yong-Yeon Cho

**Affiliations:** 1 College of Pharmacy, The Catholic University of Korea, 43, Jibong-ro, Wonmi-gu, Bucheon-si, Gyeonggi-do 420-743 Republic of Korea; 2 College of Pharmacy, Seoul National University, 1, Gwanak-ro, Gwanak-gu, Seoul 151-742 Republic of Korea; 3 Natural Medicine Research Center, Korea Research Institute of Bioscience & Biotechnology, 30 Yeongudanji-ro, Ochang-eup, Cheongwon-gun, ChungBuk 363-883 Republic of Korea

## Abstract

**Background:**

Magnolin is a natural compound abundantly found in *Magnolia* flos, which has been traditionally used in oriental medicine to treat headaches, nasal congestion and anti-inflammatory reactions. Our recent results have demonstrated that magnolin targets the active pockets of ERK1 and ERK2, which are important signaling molecules in cancer cell metastasis. The aim of this study is to evaluate the effects of magnolin on cell migration and to further explore the molecular mechanisms involved.

**Methods:**

Magnolin-mediated signaling inhibition was confirmed by Western blotting using RSK2^+/+^ and RSK2^−/−^ MEFs, A549 and NCI-H1975 lung cancer cells, and by NF-κB and Cox-2 promoter luciferase reporter assays. Inhibition of cell migration by magnolin was examined by wound healing and/or Boyden Chamber assays using JB6 Cl41 and A549 human lung cancer cells. The molecular mechanisms involved in cell migration and epithelial-to-mesenchymal transition were determined by zymography, Western blotting, real-time PCR and immunocytofluorescence.

**Results:**

Magnolin inhibited NF-κB transactivation activity by suppressing the ERKs/RSK2 signaling pathway. Moreover, magnolin abrogated the increase in EGF-induced COX-2 protein levels and wound healing. In human lung cancer cells such as A549 and NCI-H1975, which harbor constitutive active Ras and EGFR mutants, respectively, magnolin suppressed wound healing and cell invasion as seen by a Boyden chamber assay. In addition, it was observed that magnolin inhibited MMP-2 and −9 gene expression and activity. The knockdown or knockout of RSK2 in A549 lung cancer cells or MEFs revealed that magnolin targeting ERKs/RSK2 signaling suppressed epithelial-to-mesenchymal transition by modulating EMT marker proteins such as N-cadherin, E-cadherin, Snail, Vimentin and MMPs.

**Conclusions:**

These results demonstrate that magnolin inhibits cell migration and invasion by targeting the ERKs/RSK2 signaling pathway.

**Electronic supplementary material:**

The online version of this article (doi:10.1186/s12885-015-1580-7) contains supplementary material, which is available to authorized users.

## Background

Magnolin is the major component abundantly found in the dried buds of the magnolia flower, Shin-Yi, which has been traditionally used as an oriental medicine to treat nasal congestion associated with headaches, sinusitis, inflammation, and allergic rhinitis [1]. A previous study has indicated that topical application of the *Magnolia* flos (flosculous: a small budding flower) extract inhibits passive cutaneous anaphylaxis induced by anti-dinitrophenyl (DNP) IgE in rats [2]. Recent studies have demonstrated that magnolin inhibits the production of tumor necrosis factor-α (TNF-α) and prostaglandin E2 (PGE2) by inhibiting extracellular signal-regulated kinases (ERKs) [3, 4], which are key signaling molecules in the regulation of cell proliferation, transformation [5] and cancer cell metastasis [6]. Our previous results have demonstrated that magnolin targeting ERK1 (IC_50_ 87 nM) and ERK2 (IC_50_ 16.5 nM) inhibits cell transformation induced by tumor promoters such as epidermal growth factor (EGF) [5]. To date, no direct evidence regarding the inhibitory effects of magnolin on metastasis has been provided.

The 90 kDa ribosomal S6 kinases (p90RSKs: RSKs) are a family of serine/threonine kinases activated by the Ras/MEKs/ERKs signaling pathway, which responds to diverse extracellular stimuli [7]. RSK2 is a member of the RSK family and is phosphorylated at the C-terminal kinase and linker domains by ERK1/2 [8] and at the N-terminal kinase domain by phosphoinositide-dependent kinase 1 (PDK1) [9]. Activated RSK2 transduces its activation signal to various downstream target proteins including transcription and epigenetic factors [10–12], kinases [13], and scaffolding proteins such as nuclear factor of κ light polypeptide gene enhancer in B-cells inhibitor α (IκBα) [14], and regulates diverse cellular activities involved in cell proliferation, transformation and motility [15]. For instance, our previous results have demonstrated that the enhanced cAMP-dependent transcription factor 1 (ATF1) activity, caused by the epidermal growth factor (EGF)-mediated Ras/ERKs/RSK2 signaling pathway, induces cell proliferation and transformation [16]. The increased NF-κB transactivation activity, resulting from the RSK2-IκBα signaling pathway, modulates cell survival induced by the FAS-mediated death signaling pathway [13]. A recent report demonstrates that RSK2 promotes the invasion and metastasis of head and neck squamous cell carcinoma cells in humans [17]. Therefore, the Ras/ERKs/RSK2 signaling axis may be a key signaling pathway in the regulation of cell proliferation and transformation, and in cancer cell metastasis.

Nuclear factor-κB (NF-κB) is a ubiquitous nuclear transcription factor composed of p65 (Rel A), p68 (Rel B), p75 (c-Rel), p50 and p52 [18]. In the absence of cellular stimulation, NF-κB is located in the cytoplasm and forms a complex with specific inhibitors of NF-κB (IκBs). Upon cell stimulation by growth factors and proinflammatory cytokines, IκBα is phosphorylated by IκBα kinase (IKK), leading to ubiquitination and degradation [19]. Following degradation of IκBα, NF-κB translocates to the nucleus and effects the expression of genes involved in cell proliferation, invasion and metastasis [19]. Recently, we identified an alternative signaling pathway regulating NF-κB activation, in which RSK2 phosphorylates IκBα at Ser32, promoting the ubiquitination-mediated degradation of IκBα [20]. Due to the fact that ERK1 and 2 are direct upstream kinases of RSK2 [8], targeting ERK1/2 with small molecules may be the focus in the development of a drug acting as a metastatic inhibitor.

The mitogen-activated protein kinase (MAPK) family is comprised of three subfamilies including ERKs, p38 kinases and c-Jun N-terminal kinases (JNKs), which play a key role in the regulation of cellular responsiveness by the diverse extracellular stimuli such as growth factors, peptide hormones, and environmental stressors such as ultraviolet light [13, 21, 22]. The ERKs/RSK2 signaling axis plays a pivotal role in cell proliferation, differentiation, survival, and transformation [8, 10, 13, 15, 21], in addition to cell migration through the induction of matrix metalloproteinases (MMPs), which are the enzymes that degrade the extracellular matrix, such as collagen and gelatin, to facilitate the metastasis of cancer cells [6]. Recently, our research group found that magnolin, a major component of *Magnolia* flos (Shin-Yi) that has been traditionally used as an oriental medicine to treat headaches, nasal congestion and inflammatory reactions [23], inhibits the Ras/ERKs/RSK2 signaling axis by targeting the active pocket of ERK1 and ERK2 with IC_50_ values of 87 nM and 16.5 nM, respectively [5]. Furthermore, we found that AP-1 and NF-κB transactivation activities were downregulated by the inhibition of ERK1/2-mediated RSK2 activity [5, 20], suggesting that magnolin may suppress the gene expression of *Cox-2*, an enzyme that plays an important role in cancer cell proliferation, motility and metastasis [24]. Generally, metastasis is complicated multiple biological processes including 1) loss of adhesion involved during epithelial to mesenchymal transition (EMT), 2) increased motility and invasiveness to achieve intravasation, 3) circulation through blood vessels and lymph nodes, and 4) attachment to blood vessels followed by extravasation [25]. Eventually, the metastatic cancer cells succeed in colonizing on distant organ tissues, which causes more than 90 % of cancer deaths [26]. However, the molecular mechanisms behind magnolin-mediated cell migration and invasion are not yet clearly understood.

## Methods

### Reagents and antibodies

Chemical reagents such as NaCl, Tris, sodium dodecyl sulfate (SDS) and buffer preparations were purchased from Sigma-Aldrich chemical Co. (St. Louis, MO, USA). Recombinant EGF was purchased from BD Sciences (San Jose, CA, USA). Antibodies against phospho-IκBα, total-IκBα, N-cadherin, β-actin, MMP-2, MMP-9, E-cadherin, COX-2, and total-RSK2 were purchased from Cell Signaling Technology (Beverly, MA, USA), Santa Cruz Biotechnology (Santa Cruz, CA, USA) and Thermo Fisher Scientific Inc. (Waltham, MA, USA). Cell culture media and other supplements were purchased from Life Science Technology (Rockville, MD, USA) and Corning (Corning, NY, USA).

### Magnolin

Magnolin was extracted from the dried flower buds of *Magnolia fargesii* in accordance with the method established by Lee *et al*., (Korea Patent # 10-0321212-0000) [27] and confirmed a purity of >99.0 % using high-performance liquid chromatography (HPLC), which was generously provided by Dr. SR Oh of the Korean Research Institute of Bioscience and Biotechnology (KRIBB). Magnolin was prepared as a stock solution (100 mM: 1000X) by dissolving in DMSO obtained from Sigma-Aldrich Co. LLC., (St. Louis, MO, USA), after which it was aliquoted and stored at −20 °C. The magnolin was freshly diluted in DMSO before utilization, and the cells were treated upon medium exchange with magnolin premixed cell culture medium, in which the DMSO concentration did not exceed 0.1 % of the total volume.

### Cell culture and transfection

JB6 Cl41 cells purchased from ATCC were cultured with 5 % FBS-MEM, and RSK2^+/+^ and RSK2^−/−^ mouse embryonic fibroblasts (MEFs) were cultured with 10 % FBS-DMEM, containing penicillin/streptomycin, at 37 °C in a 5 % CO_2_ incubator. All animal experimental protocols were approved by the Institutional Animal Care and Use Committee at the Catholic University of Korea (approval number: 2014–0046). A549 and NCI-H1975 human lung cancer cells, purchased from ATCC, were cultured with 10 % FBS-F12K and 10 % FBS-RPMI 1640, respectively, according to the guidelines of Institutional Laboratory Safety. The cells were maintained by passage at 80-90 % confluence, and the media was changed every other day. Transfection of the various expression vectors was carried out using jetPEI (Polyplus-Transfection Inc., New York, NY, USA) according to the manufacturer’s protocol.

### Cell migration and invasion assay

JB6 Cl41 (7 × 10^4^), A549 (7 × 10^4^) and NCI-H1975 (7 × 10^4^) cells, and RSK2^+/+^ (7 × 10^4^) and RSK2^−/−^ (7 × 10^4^) MEFs were seeded into culture-inserts (ibidi GmbH, Martinsried, Germany) and cultured overnight. The cells were treated with mitomycin-C (10 μg/ml) for 2 h, and the culture-inserts were removed to offer a cell-free gap. The cells were treated with the indicated doses of magnolin either in the presence or absence of EGF for 12 or 24 h, and cell migration was observed under a light microscope. The migrated area was measured using the Image J computer software program (v. 1.45). To measure the magnolin effect on cancer cell invasion, a matrigel-coated invasion chamber (Corning Incorporated, Coring, NY, USA) was used. Briefly, A549 or NCI-H1975 (2.5 × 10^4^) cells were seeded into an insert chamber with FBS-free media supplemented with the indicated doses of magnolin, and cultured in 24-well plates supplemented with complete media for the appropriate time period. The cells were fixed with 4 % formaldehyde, permeabilized with methanol and stained with crystal violet. The stained cells were observed under a light microscope and those that had migrated were counted.

### Gelatin zymography

MMP-2 and −9 activities were evaluated by gelatin zymography using the cell culture supernatants. Briefly, A549 cells (4 × 10^5^) were seeded into 60 mm dishes, cultured and treated with the indicated doses of magnolin for 24 h. The culture supernatants were harvested, and 20 μg of protein from each sample were loaded on a polyacrylamide gel containing 0.2 % gelatin. The gel was washed with 2.5 % Triton X-100 buffer for 20 min, and then incubated for 24 h at 37 °C in renaturing buffer [50 mM Tris-Cl (pH 7.5), 10 mM CaCl_2_, 1 μM ZnCl_2_, 0.01 % NaN_3_]. The gels were stained with Coomassie Brilliant Blue and destained in methanol/acetic acid.

### Immunocytofluorescence (ICF)

A549 cells (6 × 10^4^) were seeded into 4-chamber slides, cultured and treated with the indicated doses of magnolin for 24 h. The cells were fixed with 4 % formalin, blocked in 1 % BSA/Tween-20/1X PBS at room temperature for 1 h, and hybridized with anti-N-cadherin primary and Flamma Fluors 552- or Alexa-488-conjugated secondary antibodies (BioActs, Incheon, Gyeonggi-do, Korea). The slides were mounted with Fluoroshield^TM^-DAPI (Sigma-Aldrich, St. Louis, MO, USA). The N- and E-cadherin protein levels were visualized under a LSM 710 laser scanning confocal microscope (Carl Zeiss, Oberkochen, Germany).

### Real-time PCR (RT-PCR)

A549 cells (5 × 10^5^) were seeded into 60 mm dishes, cultured overnight and treated with the indicated doses of magnolin for 24 h. Total RNA was extracted using Trizol (Invitrogen, Carlsbad, CA, USA), and quantitative gene expression levels of MMP-2 and −9 were measured by real-time polymerase chain reaction (PCR) using a specific primer set, MMP-2 (Hs01548727_m1) and MMP-9 (Hs00234579_m1), a GAPDH specific real-time primer set (4352934E), and a TaqMan RNA-to-C_T_ 1-step kit (applied Biosystems, Foster City, CA, USA) according to the manufacturer’s recommended protocol. The C_T_ values of MMP-2 and MMP-9 RNA expression were normalized to the C_T_ values of GAPDH as an internal control to ensure equal RNA utilization.

### Reporter gene assay

JB6 Cl41 (2 × 10^4^) cells stably expressing an *NF-κB- or Cox-2-promoter* luciferase reporter plasmid, and A549 cells (2 × 10^4^) cells stably expressing an *MMP-2* or an *MMP-9 promoter* luciferase reporter plasmid were seeded into a 24-well plate and cultured overnight. The cells were starved for 16 h, pretreated with the indicated doses of magnolin for 30 min, and then co-treated with EGF (10 ng/ml) at the indicated doses of magnolin for 24 h. The cells were disrupted, and the firefly luciferase activities were measured using a VIXTOR X3 fluoro/luminometer (Perkin Elmer Inc., Waltham, MA, USA).

### Western blotting

Samples containing equal amounts of proteins as indicated were resolved by SDS polyacrylamide gel electrophoresis and transferred to PVDF membranes. The membranes were blocked with 5 % skimmed milk/1X PBS/0.5 % Tween 20 at room temperature for 1 h, and hybridized with the specific primary and HRP-conjugated secondary antibodies as indicated. The proteins were visualized by an enhanced chemiluminescence (ECL) detection system (Amersham Bioscience Corp., Piscataway, NJ, USA).

## Results

### Magnolin inhibits NF-κB transactivation activity

**Fig. 1:**
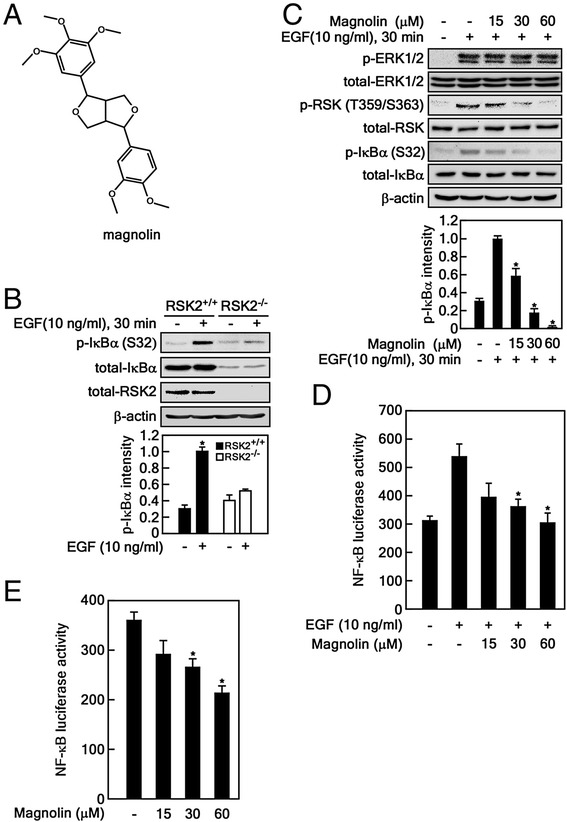
Magnolin inhibits NF-κB transactivation activity. **a** Chemical structure of magnolin. **b** RSK2 mediates IκBα phosphorylation at Ser32. RSK2^+/+^ and RSK2^−/−^ MEFs were used to visualize the phosphorylation of IκBα induced by EGF stimulation using the specific antibodies as indicated. **c** Magnolin inhibits EGF-induced IκBα phosphorylation at Ser32. JB6 Cl41 (1 × 10^6^) cells were seeded, starved, and treated with magnolin in the presence or absence of EGF. The specific protein levels were visualized by Western blotting using the specific antibodies as indicated. **b**–**c** β-actin was used as an internal control to verify equal protein loading. **d**–**e** Magnolin inhibits EGF-induced NF-κB transactivation activity. JB6 Cl41 cells stably expressing *NF-κB* luciferase reporter plasmid were seeded, cultured, starved, and stimulated with EGF and the indicated doses of magnolin (**d**), or treated with the indicated doses of magnolin under normal culture conditions for 24 h (**e**). Firefly luciferase activity was measured as described in “Materials and Methods”. **b**–**e** Data are presented as the mean ± S.D. of values from triplicate experiments, and statistical significance was determined using the Student’s *t*-test (*, *p* < 0.05)

NF-κB activation plays a key role in cell migration [28]. Our previous results have demonstrated that RSK2 induced the proteasomal degradation of IκBα by phosphorylation at Ser32, resulting in activation of NF-κB transactivation activity [20], and magnolin, a natural compound abundantly found in Shin-Yi (Fig. 1a and Additional file 1: Figure S1), inhibited ERK1 and ERK2 [5]. Moreover, RSK2 activity is regulated by ERK1/2 [8]. Therefore, we hypothesized that magnolin may inhibit cell migration. To examine this hypothesis, we used RSK2^+/+^ and RSK2^−/−^ MEFs (Fig. 1b) and confirmed that RSK2 deficiency abrogated EGF-induced IκBα phosphorylation at Ser32 (Fig. 1b). Notably, magnolin treatment suppressed EGF-induced IκBα phosphorylation at Ser32 in a dose-dependent manner, and 60 μM magnolin totally abolished EGF-induced IκBα phosphorylation at Ser32 through the inhibition of RSK phosphorylation (Fig. 1c). The phosphorylation status at Ser32 and Ser36 of IκBα served as a degron motif for the SCF-βTrCP ubiquitin E3 ligase complex, which resulted in the degradation of IκBα [19], resulting in NF-κB nuclear localization [18]. Our results strongly suggest that magnolin-mediated inhibition of ERK1/2 activity may inhibit RSK2-mediated NF-κB transactivation activity. To determine whether magnolin inhibits NF-κB transactivation activity, we starved the cells and treated them with EGF. We found that EGF treatment induced NF-κB transactivation activity (Fig. 1d). Notably, magnolin treatment suppressed EGF-induced NF-κB transactivation activity in a dose-dependent manner (Fig. 1d). Further, we found that NF-κB transactivation activity was inhibited under normal cell culture conditions by magnolin treatment in a dose-dependent manner (Fig. 1e). These results demonstrate that magnolin inhibited ERK1/2 activity, which resulted in the suppression of RSK2/NF-κB signaling by inhibiting IκBα phosphorylation at Ser32.

### Magnolin suppresses EGF-induced cell migration in JB6 Cl41 cells

**Fig. 2:**
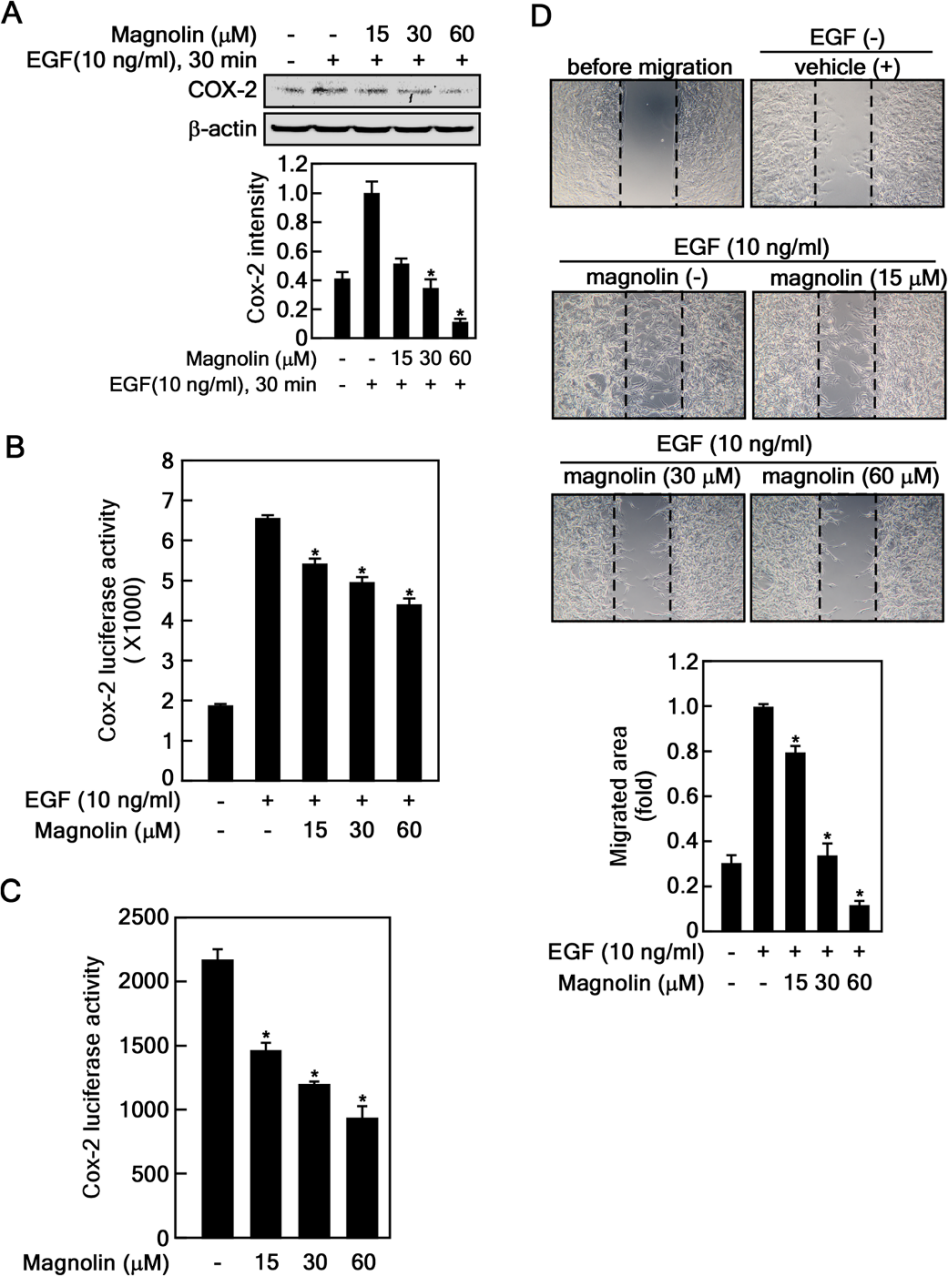
Magnolin suppresses EGF-induced cell migration in JB6 Cl41 cells. **a** Magnolin inhibits COX-2 protein levels. JB6 Cl41 cells were starved, treated with the indicated doses of magnolin and EGF for 30 min, and then the COX-2 protein level was visualized by Western blotting. β-actin was used as an internal control to verify equal protein loading. **b**–**c** Magnolin suppressed EGF-induced *Cox-2* promoter activity. JB6 Cl41 cells stably expressing *Cox-2-promoter* luciferase reporter plasmid were seeded, cultured, starved and the luciferase activities analyzed for the *Cox-2* promoter activity by combination treatment with EGF and magnolin as indicated (**b**), or the Cox-2 gene expression was analyzed with magnolin treatment under normal cell culture conditions for 24 h (**c**) as described in “Materials and Methods”. **d** Magnolin inhibits EGF-induced cell migration. JB6 Cl41 cells were seeded into culture-inserts and cultured overnight. Cell proliferation was stopped by mitomycin-C treatment for 2 h, and culture inserts were removed to offer a cell-free gap. The cells were treated with EGF or EGF and magnolin for 24 h, cell migration was observed under a microscope, and then the migrated area was measured using the Image J computer software program (v. 1.45). **a**–**d** Data are presented as the mean ± S.D. of values from triplicate experiments, and statistical significance was determined using the Student’s *t*-test (*, *p* < 0.05)

Cyclooxygenase-2 (COX-2) is one of the downstream targets of NF-κB and plays a key role in inflammation, tumorigenesis, angiogenesis, invasion and migration [29]. The cytosolic NF-κB heterodimer complex, composed of p65 (Rel A), p50 and IκBα, was activated by various stimuli including environmental stresses, cytokines and growth factors [30]. The EGF-mediated signaling pathway induces COX-2 mediated inflammation and cell migration through RSK2-mediated IκBα destabilization [20, 29]. Recently, we found that magnolin targeted ERK1 and 2, inhibited their activity with an approximate IC_50_ of 87 nM and 16.5 nM, respectively, and suppressed cell proliferation and neoplastic cell transformation *ex vivo* [5]. Thus, we hypothesized that magnolin may inhibit the proinflammatory regulator COX-2, and ultimately wound healing. We found that EGF-induced COX-2 protein levels were suppressed by magnolin treatment in a dose-dependent manner (Fig. 2a). Notably, the *Cox-2* promoter activity induced by EGF was abolished by magnolin treatment (Fig. 2b). Additionally, the *Cox-2* promoter activity was also suppressed by magnolin treatment under normal cell culture conditions (Fig. 2c). Further, we found that JB6 Cl41 cell migration enhanced by EGF treatment was dramatically suppressed by magnolin treatment in a dose-dependent manner (Fig. 2d). Our previous results have demonstrated that magnolin suppressed RSK2 activity by inhibiting ERK1/2-mediated-RSK2 phosphorylation at a linker region and a C-terminal kinase domain [5]. Taken together, our results demonstrate that magnolin inhibited ERK1/2/RSK2 signaling-mediated IκBα phosphorylation at Ser32, resulting in the inhibition of NF-κB activation and cell migration.

### Magnolin inhibits migration and invasion of human lung cancer cells

**Fig. 3:**
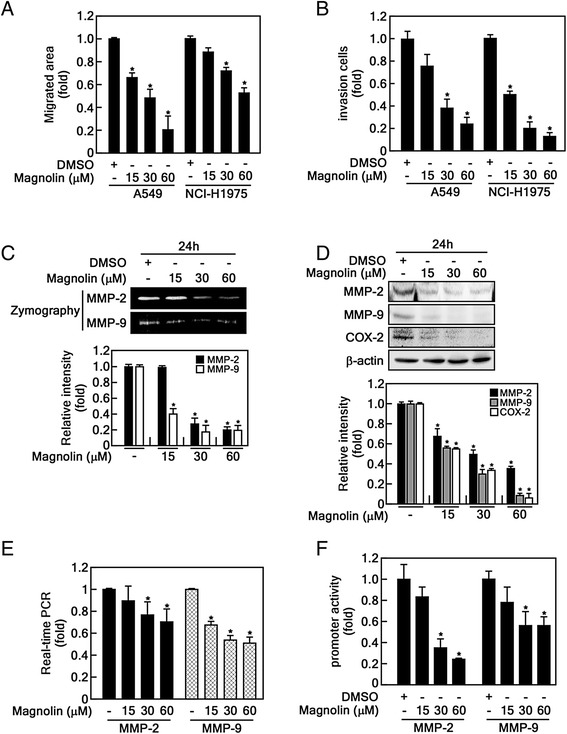
Magnolin inhibits migration and invasion of human lung cancer cells. **a** Magnolin inhibits cell migration. A549 or NCI-H1975 cells were seeded into culture-inserts and treated with mitomycin-C. The culture-inserts were removed, the cell migration was measured using the Image J computer software program (v. 1.45), followed by treatment with the indicated doses of magnolin for 24 h. **b** Magnolin inhibits cancer cell invasion. A549 and NCI-H1975 cells were seeded into the inserts of Boyden chambers and cultured overnight. The cells were treated with the indicated doses of magnolin, and cell invasion was allowed for 24 h. The migrated cells were stained with crystal violet, observed and counted under an inverted microscope. **c** Magnolin suppresses the activity of MMP-2 and MMP-9. A549 cells (5 × 10^5^) were seeded, cultured, and treated with the indicated doses of magnolin for 24 h. The cultured media was harvested for zymography as described in “Materials and Methods”. **d** Magnolin reduces the protein levels of MMP-2 and −9. MMP-2 and −9 protein levels from (**c**) were visualized by Western blotting using the specific antibodies as indicated. β-actin was used as an internal control to verify equal protein loading. **e** Magnolin inhibits the gene expression of MMP-2 and −9. The MMP-2 and −9 mRNA levels from the cells in (**c**) were analyzed by real-time PCR as described in “Materials and methods”. **f** Magnolin suppresses the promoter activities of MMP-2 and −9. A549 cells stably expressing *MMP-2* or *MMP-9* promoter luciferase reporter plasmid were seeded, cultured, and treated with the indicated doses of magnolin for 24 h. Firefly luciferase activity was measured as described in “Materials and Methods”. **a**–**f** Data are presented as the mean ± S.D. of values from triplicate experiments, and statistical significance was determined using the Student’s *t*-test (*, *p* < 0.05)

Our previous study indicated that magnolin inhibited cell proliferation of A549 cells [5], a human lung cancer cell line harboring metastatic capabilities such as invasion and migration [5, 31]. Therefore, we hypothesized that magnolin may suppress the metastatic capabilities of lung cancer cells such as A549 and NCI-H1975 cells. To examine this hypothesis, we conducted a wound healing assay. We found that cell migration into a cell-free gap was inhibited by magnolin treatment approximately 50 % ~ 80 % at 30 μM and 60 μM in A549 cells and 50 % at 60 μM magnolin in NCI-H1975 cells (Fig. 3a and Additional file 2: Figure S2A). Moreover, we found that cancer cell invasion by the Boyden chamber assay indicated that magnolin suppressed cancer cell invasion in a dose dependent manner in both human lung cancer cells (Fig. 3b and Additional file 2: Figure S2B). Recently, the MEK inhibitor PD98059 has been shown to dramatically suppress the activities and gene expressions of MMP-2 and −9 in zymography [32–34], indicating that magnolin may modulate MMP-2 and −9 activities by suppression of gene expression. To examine these hypotheses, we collected cell culture medium and analyzed MMP-2 and −9 activities by zymography. We found that secreted MMP-2 and −9 activities were decreased by magnolin in a dose-dependent manner (Fig. 3c). Moreover, MMP-2 and −9 protein levels in the cells were decreased gradually in correlation with an increase in the magnolin concentration (Fig. 3d). These results were firmly supported by the reproducible COX-2 protein levels that magnolin inhibited through the suppression of *Cox-2* gene expression (Fig. 2a–c). To examine the casual reasons for the decrease in MMP-2 and −9 protein levels, we conducted real-time PCR using MMP-2 and MMP-9 specific primer sets and found that the gene expression of MMP-2 and −9 was inhibited by magnolin treatment (Fig. 3e). Simultaneously, we found that the promoter activities of MMP-2 and −9 were suppressed by magnolin treatment in a dose-dependent manner (Fig. 3f). These results demonstrate that magnolin suppresses cell migration and invasion in human lung cancer cells.

### Magnolin inhibits epithelial-to-mesenchymal transition

**Fig. 4:**
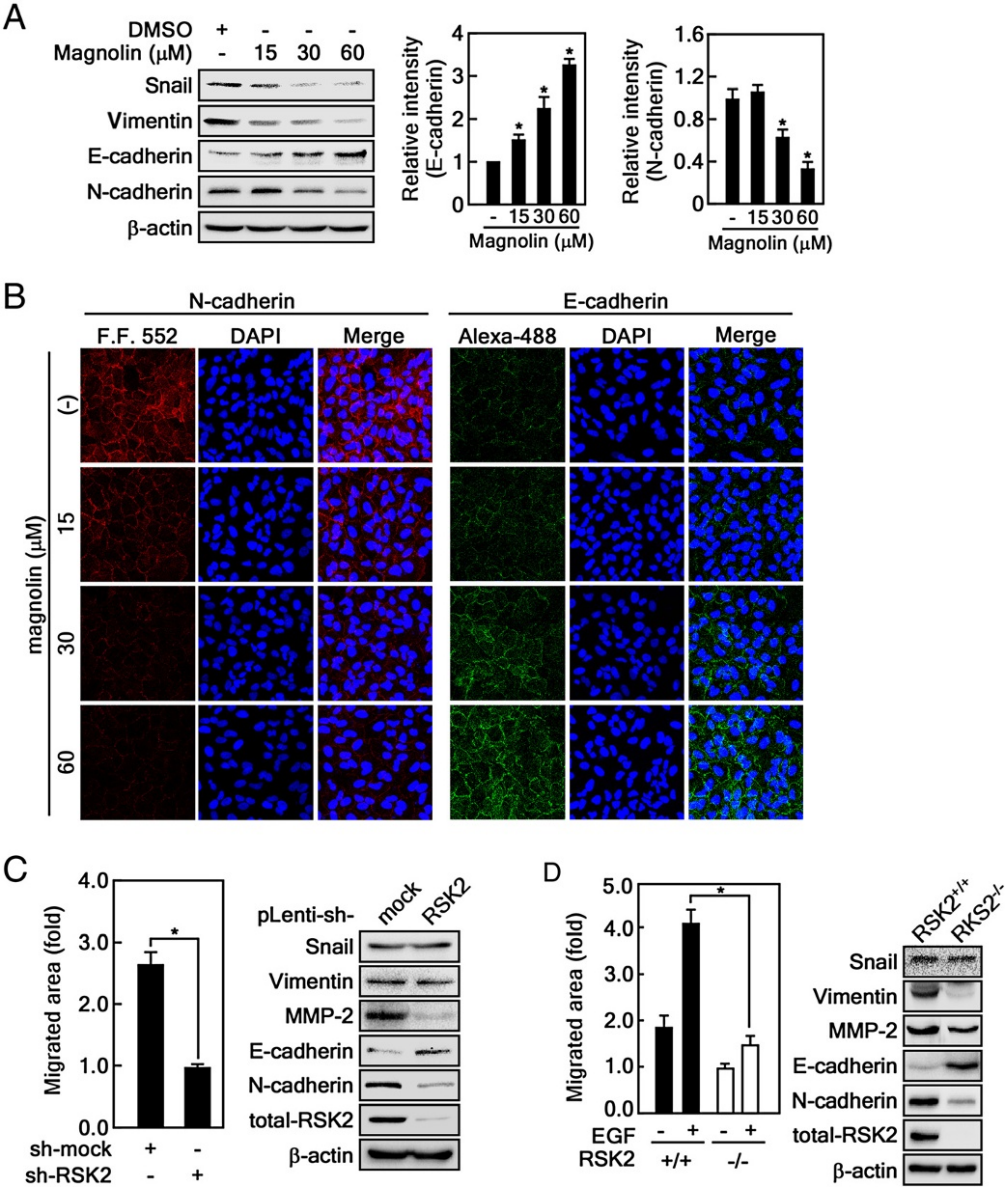
Magnolin inhibits epithelial-to-mesenchymal transition. **a** Effects of magnolin on the protein levels of epithelial-mesenchymal marker proteins. A549 cells were seeded, cultured, treated with the indicated doses of magnolin for 24 h. The proteins were extracted and visualized by Western blotting using the specific antibodies as indicated. **b** Effects of magnolin on the N- and E-cadherins by immunocytofluorescence analysis in A549 cells. The protein levels of N- and E-cadherins altered by magnolin treatment were visualized by immunocytofluorescence as described in “Materials and Methods”. **c** RSK2 knockdown effects on the protein levels of EMT marker proteins. *Graph*, knockdown of RSK2 inhibits cell migration in A549 cells. A549 cells stably expressing sh-mock or sh-RSK2 were seeded into culture-inserts and treated with mitomycin-C for 2 h. The culture-inserts were removed, and the cell migration was measured 24 h later using the Image J computer software program (v. 1.45). *Panels*, A549 cells stably expressing sh-mock or sh-RSK2 were seeded, cultured and harvested. The protein levels were visualized by Western blotting using the specific antibodies as indicated. **d** RSK2 deficiency suppresses cell migration and EMT marker protein levels. *Graph*, RSK2^+/+^ mesenchymal and RSK2^−/−^ MEFs were seeded into culture-inserts and treated with mitomycin-C for 2 h. The culture-inserts were removed, treated with 10 ng/ml EGF and cell migration was allowed for 24 h. The cell migration was observed and measured using the Image J computer software program (v. 1.45). *Panels*, RSK2^+/+^ and RSK2^−/−^ MEFs were seeded, cultured and harvested. The proteins were extracted and visualized by Western blotting using the specific antibodies as indicated. **a**, **c** and **d** β-actin was used as an internal control to verify equal protein loading. Data are presented as the mean ± S.D. of values from triplicate experiments, and statistical significance was determined using the Student’s *t*-test (*, *p* < 0.05)

In order to metastasize, cancer cells are required to change their behavior [25]. Colonized cancer cells in tumors dissociate by transitioning from epithelial cells into cells that have mesenchymal properties [35]. The processes are mediated by the alteration of adhesion molecules such as E-cadherin, an epithelial marker, and N-cadherin, a mesenchymal marker [35]. Our results demonstrate that magnolin suppressed cell migration and invasion (Figs. 2 and 3). These results strongly suggest that magnolin may modulate epithelial-to-mesenchymal transition. To examine this hypothesis, we conducted Western blotting and found that magnolin enhanced E-cadherin and suppressed N-cadherin protein levels in A549 lung cancer cells (Fig. 4a). Notably, the protein levels of EMT marker proteins such as Snail and Vimentin were decreased by magnolin treatment in a dose-dependent manner (Fig. 4a). Immunocytofluorescence data clearly demonstrated that magnolin abolished the epithelial-to-mesenchymal transition in A549 lung cancer cells (Fig. 4b). However, we could not find a cell morphological change induced by magnolin (Additional file 3: Figure S3). To analyze the RSK2 involvement in EMT, we utilized RSK2 knockdown cells using RSK2 sh-RNA and found that RSK2 knockdown suppressed wound healing of A549 lung cancer cells (Fig. 4c, *graph,* and Additional file 4: Figure S4A). Interestingly, RSK2 knockdown by RSK2 sh-RNA suppressed N-cadherin and MMP-2 protein levels, and enhanced E-cadherin protein levels without dramatic alteration of Snail and Vimentin protein levels (Fig. 4c, *Western blotting*), but with a morphological change of A549 cells (Additional file 4: Figure S4B). Importantly, RSK2 deficiency abrogated the cell migration induced by EGF compared with RSK2^+/+^ MEFs (Fig. 4d, *graph*, and Additional file 4: Figure S4C). The EMT marker proteins including Vimentin, MMP-2, and N-cadherin were highly detected in RSK2^+/+^ MEFs, with E-cadherin being hardly detected, and Snail was slightly decreased in RSK2^−/−^ MEFs (Fig. 4d, Western blotting). These results strongly support the notion that magnolin suppresses cell migration and invasion by targeting the ERKs/RSK2 signaling pathway.

## Discussion

Oriental medicinal herbs contain many useful compounds, and have been widely used to identify novel compounds that may have therapeutic value in the treatment of human diseases. For instance, myricetin and quercetin from dietary herbs and epigallocatechin gallate from green tea inhibit cell proliferation and transformation [36], highlighting the importance of efforts to identify natural compounds that inhibit the ERKs/RSKs signaling pathway, while suppressing the MAPK pathway in a non-toxic manner [37]. Early buds of the magnolia flower are an oriental medicinal herb and traditionally used to treat inflammation-mediated human diseases including empyema, nasal congestion, sinusitis and allergic rhinitis [1]. Recent reports have provided evidence that magnolin inhibits the expression of cell adhesion molecules including intercellular adhesion molecule-1 and vascular cell adhesion molecule-1 [38]. However, although magnolin has shown diverse effects on human diseases, the molecular targets of magnolin had not yet been identified. Recently, our research group found that ERK1 and ERK2 are the molecular targets of magnolin, which inhibited their kinase activity with IC_50_ values of 87 nM and 16.5 nM, respectively, by competing with ATP in an active pocket [5]. Furthermore, our previous results have demonstrated that ERK1/2-mediated RSK2 activation modulates NF-κB activity by phosphorylation of IκBα at Ser32 [20]. These results provide us with the rationale to consider that magnolin may show effectiveness on cell migration and cancer metastasis. In pancreatic cancer, the NF-κB signaling pathway plays an important role in EMT and metastasis [39, 40]. Moreover, NF-κB activation induces classical EMT marker changes and the promotion of cell migration and invasion [41], indicating that ERK/RSK2/NF-κB signaling may play a key role in cell migration and invasion. Our results support the notion that RSK2 activity modulates NF-κB activity (Fig. 1c and d). Importantly, the knockdown and knockout of RSK2 attenuated cell migration (Fig. 4c and d), which was similarly observed upon magnolin treatment of A549 and NCI-H1975 lung cancer cells (Fig. 3a and b). These results demonstrate that ERK inhibition by magnolin suppresses RSK2-mediated NF-κB activity, resulting in suppression of cell migration and invasion in cancer cells.

The cellular program called EMT is accompanied by profound changes in cell characteristics that enable the epithelial cells to detach from tight junctions, change the cell’s shape and polarity, delaminate, and migrate [42]. A great number of growth factors and signaling pathways have been associated with EMT induction, including EGF through the JAK pathway and the ERK/MAPK signaling pathway [43, 44]. Previous reports have indicated that coffee or chlorogenic acid abolished CT-26 metastasis to the lung by blocking ERK/AP-1 and ERK/NF-κB signaling pathways [45]. Our results demonstrate that EGF stimulation induces cell migration in JB6 Cl41 cells (Fig. 2d). The role of RSK2 in EGF-induced cell migration was confirmed using RSK2^+/+^ and RSK2^−/−^ MEFs, that RSK2 deficiency abrogated EGF-induced wound healing (Fig. 4d). In A549 cancer cells, we further confirmed that knockdown of RSK2 using RSK2 sh-RNA suppressed cell migration (Fig. 4c). Interestingly, we found that RSK2 knockdown inhibited MMP-2 and N-cadherin and enhanced E-cadherin (Fig. 4c), however, unexpectedly, there were no significant changes observed in Snail and Vimentin (Fig. 4c). Similar results were observed with Snail, but not with Vimentin, in RSK2^+/+^ and RSK2^−/−^ MEFs (Fig. 4d). The gene expression of Snail is dependent on ERKs activity through the ERK/Fra-1/c-Jun signaling pathway [46] and the ERK/ELK-1 signaling pathway [47]. Our previous results have demonstrated that RSK2 deficiency dramatically increased total protein levels and phosphorylation sensitivity of ERK1/2 by EGF treatment [13]. Thus, we suggest that the no change in Snail is due to the reactivation of ERKs by the activation of the RSK2 feedback loop. Based on this hypothesis, it is possible to explain that magnolin inhibited cell migration and invasion by downregulating ERK-mediated Vimentin protein level by downregulating the RSK2-mediated NF-κB signaling pathway. Taken together, these results demonstrate that RSK2 mediates EGF-induced cell migration signaling through the ERKs/RSK2 signaling pathway.

The wound healing assay is a well-adapted strategy to evaluate cancer cell metastasis *ex vivo* [48]. The RSK2 function in cancer metastasis has been observed from head and neck squamous cell carcinoma (HNSCC) in cancer patients [17]. This evidence was proved by a xenograft metastasis experiment showing that knockdown of RSK2, but not RSK1, reduced the metastasis of human HNSCC cells [17]. Furthermore, RSK protein levels are important in determining whether cancer cells have the capability to metastasize. RSK1-silencing enhances *in vitro* cell migration, and human patient samples of metastatic lung cancer have lower RSK1 expression levels compared with non-metastatic cancer tissues [49]. In contrast, cancer tissue analysis from HNSCC showed a positive relationship between the metastatic ability and RSK2 protein levels [49]. Our previous results have demonstrated that total- and activated-RSK2 protein levels were observed in a human tissue array of skin cancers [8, 22]. Importantly, our *ex vivo* study demonstrated that RSK2 protein levels were more enhanced in skin cancer cells such as malignant melanoma compared with squamous cell carcinoma and premalignant immortalized cells [22]. The signaling study of RSK2 indicates that RSK2 can phosphorylate GSK3β at Ser9, resulting in enhanced cell survival from stresses such as calcium and UV irradiation [13]. Due to the fact that RSK2 is phosphorylated by ERK1 and 2, but not by p38 kinase, ERK1 and 2 inhibitors may be useful compounds to inhibit cancer cell metastasis. Magnolin is a potent natural compound, having strong inhibitory effects on ERK1 and 2 by competing with ATP in the active pockets [5], and we believe that magnolin has a potential application in human cancer prevention and metastasis.

## Conclusions

This study demonstrates that the inhibition of ERKs/RSK2 signaling by magnolin, a natural compound from dried Magnolia flos that has long been used as a traditional oriental medicine, abrogated the epithelial-to-mesenchymal transition and cell migration and invasion. The effects of magnolin on cell migration facilitated through NF-κB-mediated Cox-2 gene expression were by inhibition of ERKs/RSK2 signaling. Simultaneously, our results demonstrate that magnolin inhibited the activity of MMP-2 and MMP-9, which are critical enzymes involved in cell migration and focal adhesion. Notably, magnolin suppressed the epithelial-to-mesenchymal transition. This is the first report to demonstrate the role of magnolin as a metastatic inhibitor in lung cancer cells. As part of future research, these results may become a mechanism-driven foundation for the identification of natural compounds potentiating cancer therapeutic efficacy or cancer chemoprevention.

## Additional files

Additional file 1: Figure S1. HPLC chromatogram of magnolin. The mobile phase for HPLC consisted of solvent A, deionized water, and solvent B, 100 % methanol. The solvent gradient was as follows: 0 min, 50 % B; 50 min, 75 % B; 51 min, 98 % B, 55 min, 98 % B, and then held for 10 min before returning to the initial conditions. The flow rate was 1.0 ml/min and the injection volume was 3 μl (1 mg/ml). The chromatogram was detected at 254 nm. HPLC operating conditions: HPLC system: Agilent infinity 1260; Column: YMC pack pro C_8_ (YMC); Solvent: A, deionized water, B, methanol (gradient); Flow rate: 1.0 ml/min; Monitor: 254 nm. (TIFF 77 kb)
Additional file 2: Figure S2. Magnolin inhibits migration and invasion of cancer cells. (A) Magnolin inhibits cell migration. Human lung cancer cells, A549 and NCI-H1975, were seeded into culture-inserts and treated with mitomycin-C for 2 h. The culture-inserts were removed, and the cell migration was measured using the Image J computer software program (v. 1.45), followed by treatment with the indicated doses of magnolin for 24 h. The cell migration was photographed under an inverted microscope. (B) Magnolin inhibits cancer cell invasion. Briefly, A549 and NCI-H1975 cells were seeded onto the inserts of Boyden chambers and cultured overnight. The cells were treated with the indicated concentrations of magnolin, and cell invasion was allowed for 24 h. The migrated cells were stained with crystal violet and observed under an inverted microscope. (TIFF 5101 kb)
Additional file 3: Figure S3. Morphology of A549 cells with magnolin treatment. A549 lung cancer cells were treated with magnolin for 24 h as indicated. The cell morphology was observed under an inverted microscope. (TIFF 1528 kb)
Additional file 4: Figure S4. RSK2 regulates cell migration. (A) Knockdown of RSK2 with RSK2 sh-RNA suppresses cell migration. A549 human lung cancer cells stably expressing sh-RNA RSK2 were seeded into culture-inserts and treated with mitomycin-C for 2 h. The culture-inserts were removed and cell migration was allowed for 24 h, and then cell migration was measured using the Image J computer software program (v. 1.45). The cell migration was photographed under an inverted microscope. (B) Morphology of A549 cells by knockdown of RSK2. A549 lung cancer cells were infected with sh-RNA-mock or -RSK2, and the cell morphological change was observed under an inverted microscope. (C) RSK2 deficiency attenuates EGF-induced cell migration. RSK2^+/+^ and RSK2^−/−^ MEFs were seeded into culture-inserts and treated with mitomycin-C for 2 h. The culture-inserts were removed and cell migration was allowed with EGF treatment for 24 h. The cell migration was measured using the Image J computer software program (v. 1.45). The cell migration was photographed under an inverted microscope. (TIFF 2567 kb)
