# Supplementary figures and images for: Magnolin inhibits cell migration and invasion by targeting the ERKs/RSK2 signaling pathway
Source: BMC Cancer. 2015 Aug 8;15:576. doi: 10.1186/s12885-015-1580-7 (PMC4529708; doi:10.1186/s12885-015-1580-7)

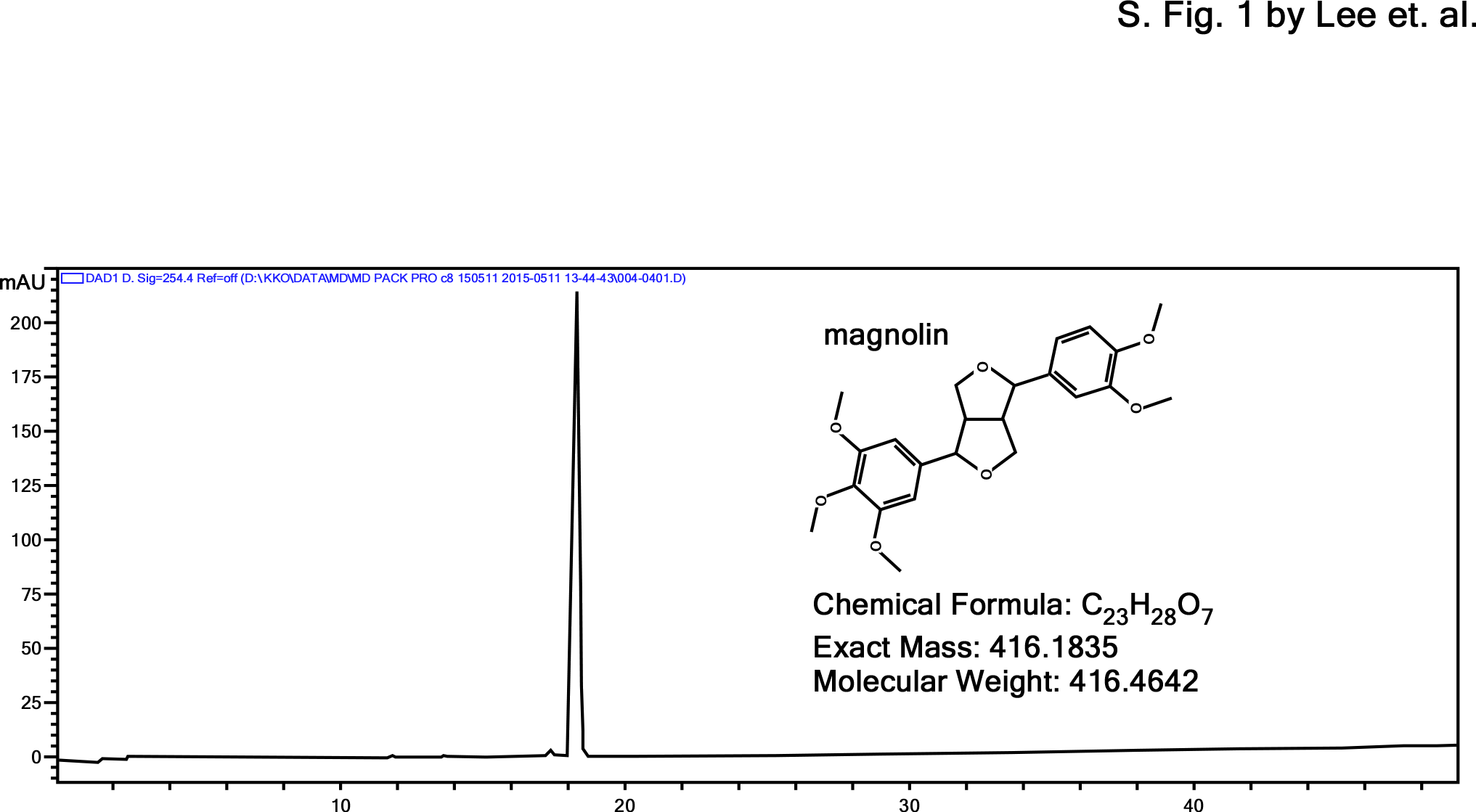

Supplement: Additional file 1: Figure S1. — HPLC chromatogram of magnolin. The mobile phase for HPLC consisted of solvent A, deionized water, and solvent B, 100 % methanol. The solvent gradient was as follows: 0 min, 50 % B; 50 min, 75 % B; 51 min, 98 % B, 55 min, 98 % B, and then held for 10 min before returning to the initial conditions. The flow rate was 1.0 ml/min and the injection volume was 3 μl (1 mg/ml). The chromatogram was detected at 254 nm. HPLC operating conditions: HPLC system: Agilent infinity 1260; Column: YMC pack pro C8 (YMC); Solvent: A, deionized water, B, methanol (gradient); Flow rate: 1.0 ml/min; Monitor: 254 nm. (TIFF 77 kb) [file 12885_2015_1580_MOESM1_ESM.tiff]

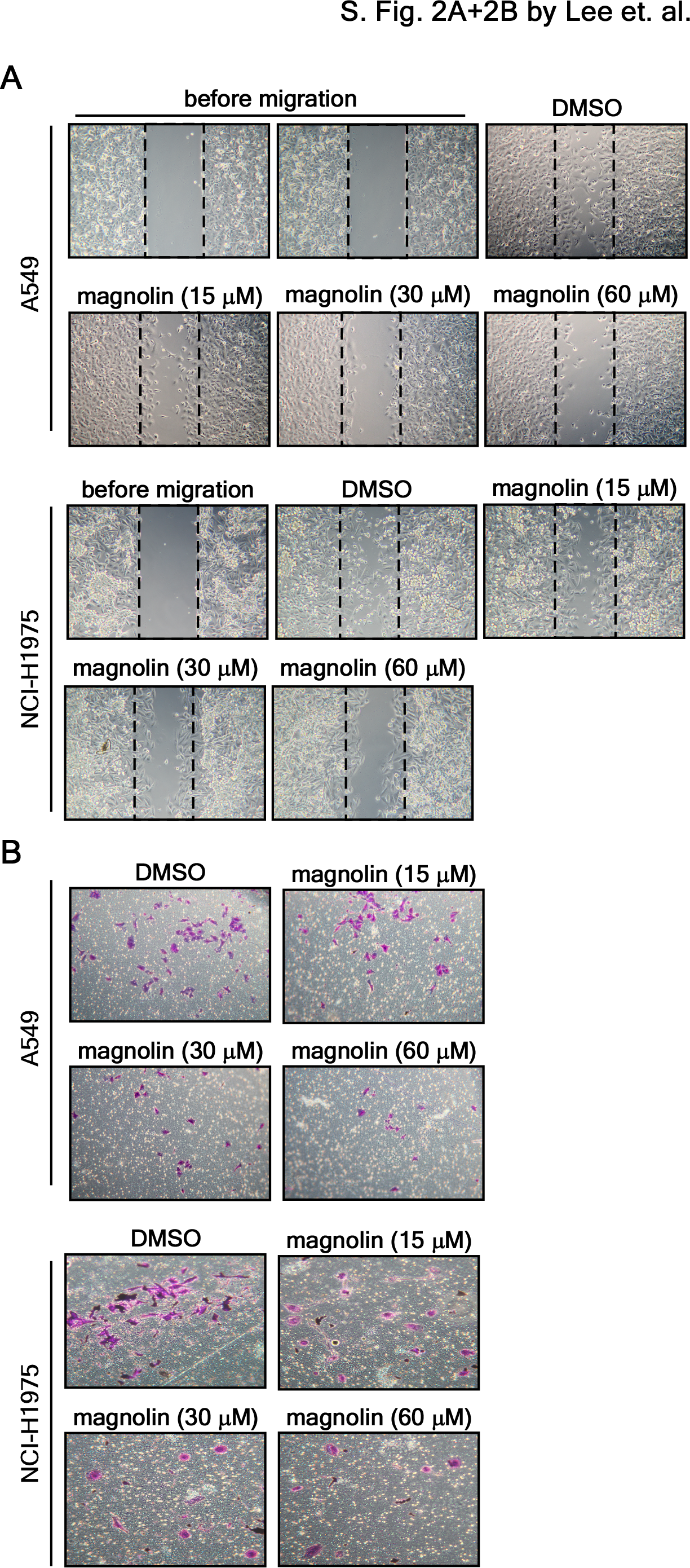

Supplement: Additional file 2: Figure S2. — Magnolin inhibits migration and invasion of cancer cells. (A) Magnolin inhibits cell migration. Human lung cancer cells, A549 and NCI-H1975, were seeded into culture-inserts and treated with mitomycin-C for 2 h. The culture-inserts were removed, and the cell migration was measured using the Image J computer software program (v. 1.45), followed by treatment with the indicated doses of magnolin for 24 h. The cell migration was photographed under an inverted microscope. (B) Magnolin inhibits cancer cell invasion. Briefly, A549 and NCI-H1975 cells were seeded onto the inserts of Boyden chambers and cultured overnight. The cells were treated with the indicated concentrations of magnolin, and cell invasion was allowed for 24 h. The migrated cells were stained with crystal violet and observed under an inverted microscope. (TIFF 5101 kb) [file 12885_2015_1580_MOESM2_ESM.tiff]

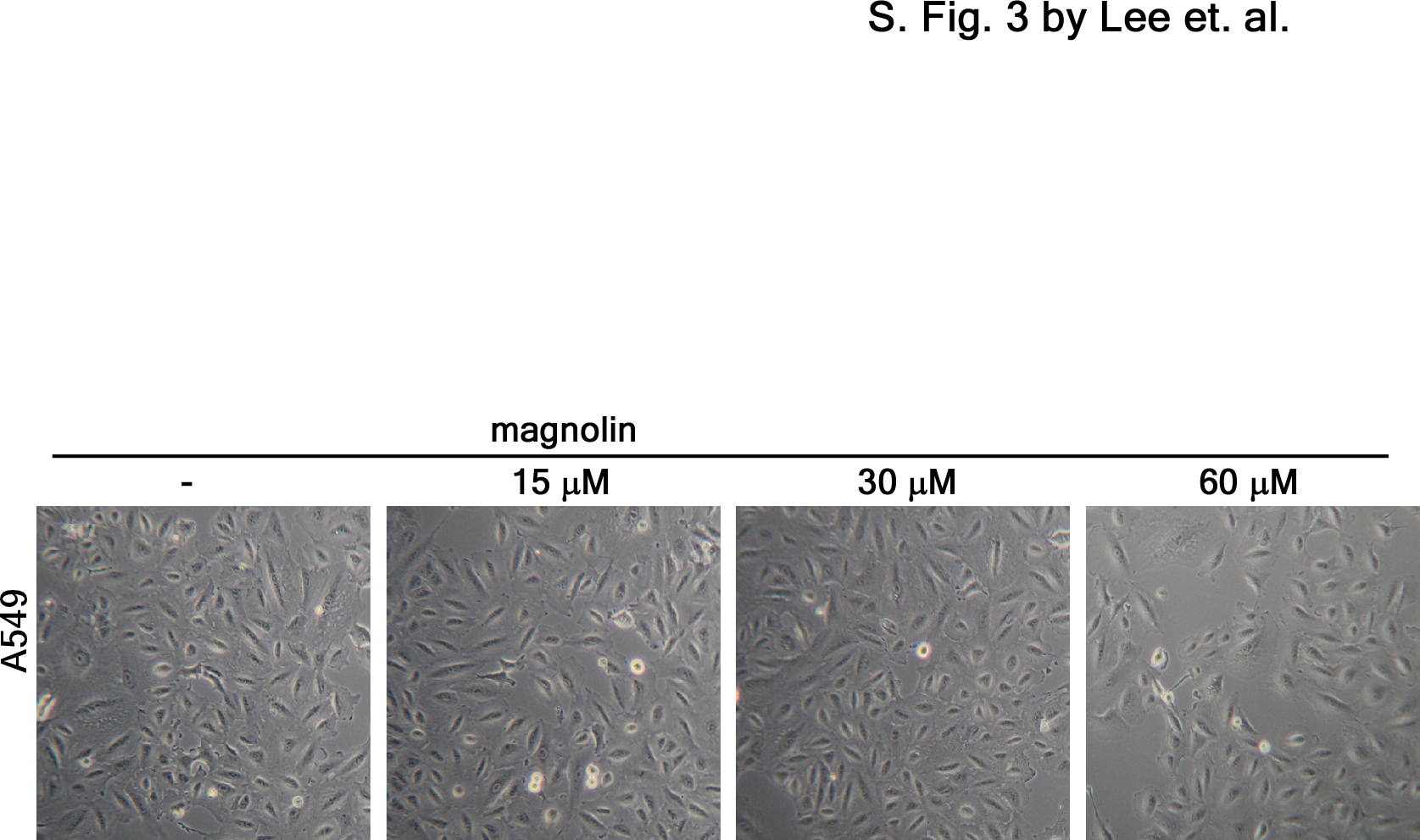

Supplement: Additional file 3: Figure S3. — Morphology of A549 cells with magnolin treatment. A549 lung cancer cells were treated with magnolin for 24 h as indicated. The cell morphology was observed under an inverted microscope. (TIFF 1528 kb) [file 12885_2015_1580_MOESM3_ESM.tiff]

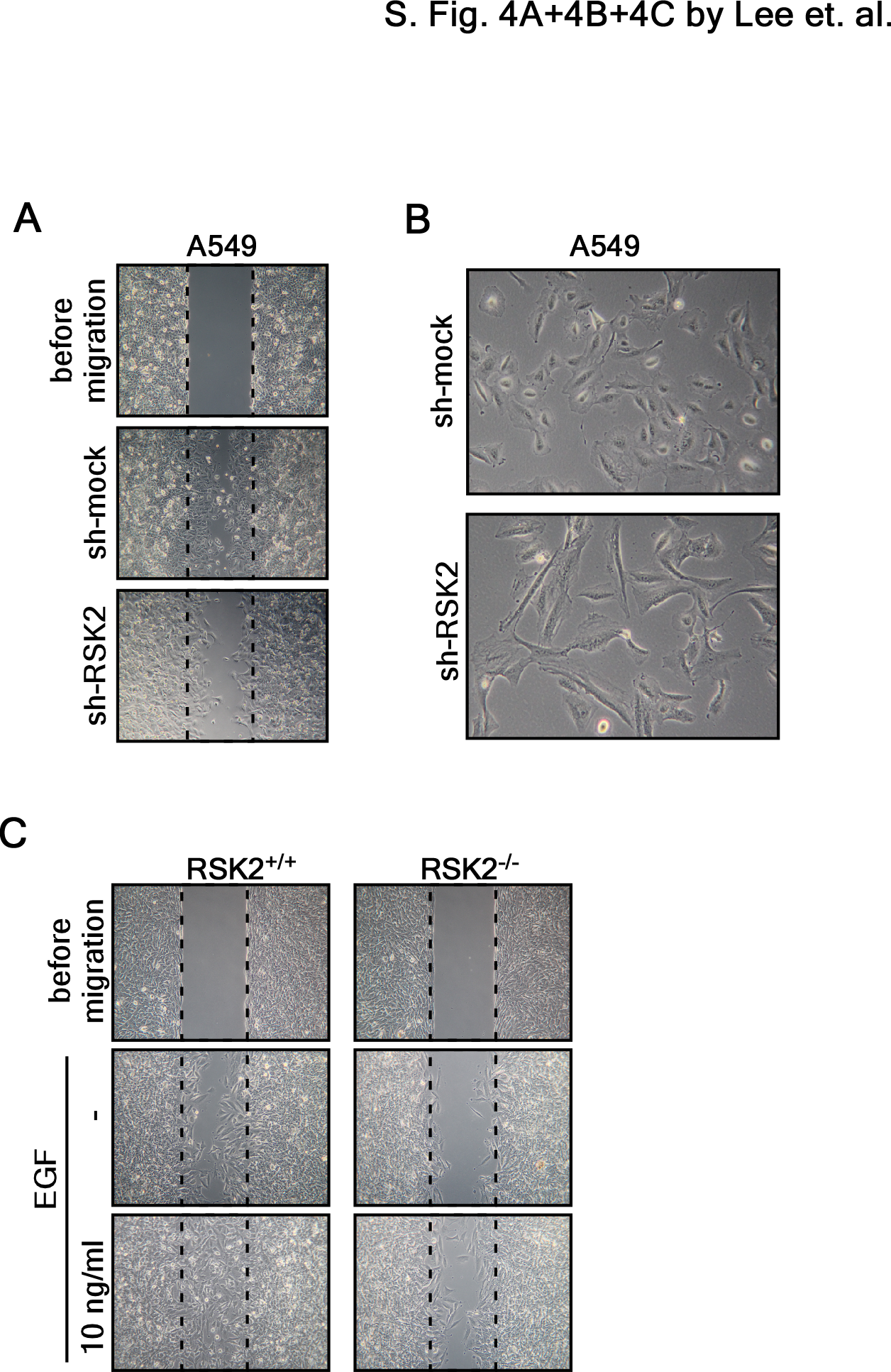

Supplement: Additional file 4: Figure S4. — RSK2 regulates cell migration. (A) Knockdown of RSK2 with RSK2 sh-RNA suppresses cell migration. A549 human lung cancer cells stably expressing sh-RNA RSK2 were seeded into culture-inserts and treated with mitomycin-C for 2 h. The culture-inserts were removed and cell migration was allowed for 24 h, and then cell migration was measured using the Image J computer software program (v. 1.45). The cell migration was photographed under an inverted microscope. (B) Morphology of A549 cells by knockdown of RSK2. A549 lung cancer cells were infected with sh-RNA-mock or -RSK2, and the cell morphological change was observed under an inverted microscope. (C) RSK2 deficiency attenuates EGF-induced cell migration. RSK2+/+ and RSK2−/− MEFs were seeded into culture-inserts and treated with mitomycin-C for 2 h. The culture-inserts were removed and cell migration was allowed with EGF treatment for 24 h. The cell migration was measured using the Image J computer software program (v. 1.45). The cell migration was photographed under an inverted microscope. (TIFF 2567 kb) [file 12885_2015_1580_MOESM4_ESM.tiff]
